# Lesion-Induced Blepharospasm: Epidemiology and Clinical Characteristics

**DOI:** 10.5334/tohm.1025

**Published:** 2025-06-09

**Authors:** Elina Myller, Rolle Halonen, Daniel T. Corp, Juho Joutsa

**Affiliations:** 1 Turku Brain and Mind Center, Clinical Neurosciences, University of Turku, Turku, Finland; 2 Neurocenter, Turku University Hospital, Turku, Finland; 3 Cognitive Neuroscience Unit, School of Psychology, Deakin University, Geelong, Australia

**Keywords:** blepharospasm, epidemiology, lesion, etiology, neuroimaging, clinical

## Abstract

**Background:**

Lesion-induced blepharospasm is considered rare. However, this information is based on a small number of heterogenous retrospective cohorts without routine neuroimaging.

**Objectives:**

To study the epidemiology and clinical characteristics of lesion-induced blepharospasm.

**Methods:**

Patients with blepharospasm with uncertain etiology prior to brain imaging were systematically searched from the electronic medical records of Turku University Hospital (1996–2022). Clinical information and imaging data were extracted from the patients’ records and re-evaluated by the investigators. Etiology of blepharospasm was evaluated by an in-depth review of the clinical information in the context of available literature on lesion-induced dystonias. The prevalence and annual incidence of blepharospasm were calculated based on the annual population count in the area.

**Results:**

The search identified 57 patients, of whom four (7.0%) were considered to have lesion-induced blepharospasm, corresponding to a population-based prevalence of 2.5 per million and annual incidence of 0.3 per million. All patients with lesion-induced blepharospasm had atypical features, which were all significantly more common than in patients with idiopathic blepharospasm (*P* < 0.05).

**Conclusions:**

Lesion-induced blepharospasm is more common than thought previously. However, all these patients showed atypical features, suggesting that brain imaging in blepharospasm can be limited to patients with atypical features only.

**Highlights:**

This study investigated the epidemiology of lesion-induced blepharospasm by systematically re-evaluating all patients with blepharospasm with uncertain etiology prior to structural brain imaging from a university hospital (1996–2022).

Our results show that lesion-induced blepharospasm is more common that thought previously (7.0% of included patients, prevalence 2.5 per million, annual incidence 0.3 per million).

All patients with lesion-induced blepharospasm showed clinical features that were considered atypical for idiopathic blepharospasm, indicating that routine brain imaging is not needed in patients with typical symptoms.

## Introduction

Blepharospasm is considered as a form of focal dystonia, characterized by excessive contractions of the orbicularis oculi and surrounding muscles in the upper cranial region [1]. According to the most recent clinical diagnostic guidelines [2], the dystonic contractions are bilateral and synchronous, and the sensitivity and specificity of the diagnosis increases with the presence of increased blinking rate, or any sensory tricks to alleviate the symptoms as in other dystonias [2,3]. Blepharospasm is defined as bilateral symmetric disorder, but the symptoms may start asymmetrically or unilaterally [4]. Blepharospasm is often accompanied by other symptoms affecting the eyes, e.g. dryness of the eyes, photophobia or pretarsal blepharospasm [2,5]. Spread of symptoms to other body regions is common, with approximately half of the patients with blepharospasm later developing dystonia in other parts of the body, further supporting the classification of blepharospasm as a type of dystonia [6,7].

Blepharospasm is the second most common form of focal dystonia after cervical dystonia [8,9,10], and it can present as isolated, or combined with other movement disorders (such as cerebellar ataxias or parkinsonism syndromes), other neurological features, or systemic disorders [11]. The etiology of blepharospasm is most often idiopathic, but it can also be secondary, caused by e.g. certain drugs, such as dopamine receptor antagonists, or focal brain lesions (e.g. vascular, neoplastic, traumatic) [1,11].

Lesion-induced blepharospasm is considered rare with an estimated percentage of only 0.4–1.6% of all patients with blepharospasm [12,13,14]. However, this assumption is based on limited information from a small number of heterogenous retrospective cohort studies [12,13,14], of which only one conducted a systematic search of the medical records [13]. In addition, as the diagnosis of blepharospasm is clinical and routine imaging is generally not recommended [15], causal lesions can go unnoticed, leading to underestimation of the true prevalence of lesion-induced blepharospasm. Furthermore, there are no previous studies investigating the population-based prevalence and incidence, or clinical characteristics of lesion-induced blepharospasm, leaving also the lack of need for routine neuroimaging in these patients uncertain.

Therefore, the aim of this study was to investigate the epidemiology and clinical characteristics of lesion-induced blepharospasm by systematically reviewing clinical information and neuroimaging data of patients with blepharospasm treated at Turku University Hospital, a regional tertiary care hospital, from 1996 to 2022.

## Methods

### Patient selection

Cases of blepharospasm were systematically searched from the electronic medical records of Turku University Hospital, one of the five regionally organized university hospitals in Finland. The records of all adult patients (age 18 or above) treated at our hospital who had received an ICD-10 diagnosis code G24.5 (blepharospasm) from the start of the electronic medical records in 1996 to 4^th^ of February 2022 were identified. The search returned 151 patients, whose medical records were screened by a neurologist (E.M.), and included cases were re-evaluated together with a movement disorder neurologist (J.J.) as needed.

**Figure 1:**
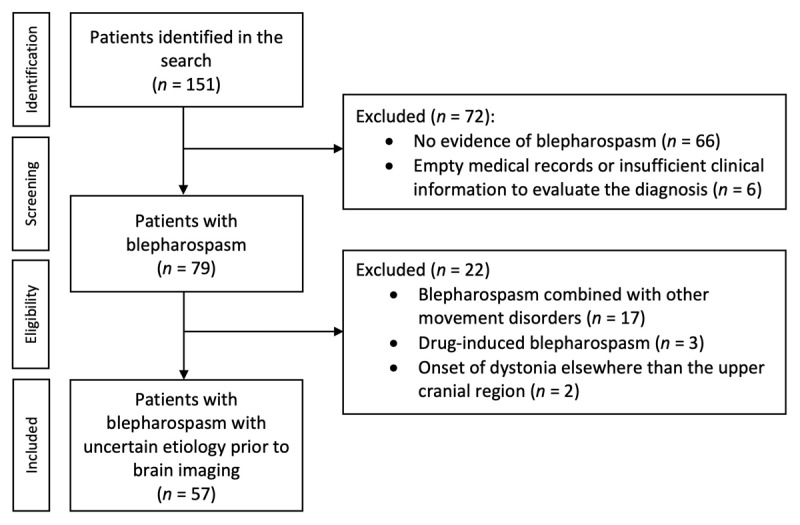
Systematic search process for patients with blepharospasm.

All patients had been evaluated at the neurology or ophthalmology outpatient clinic of Turku University Hospital. The diagnoses of blepharospasm were confirmed by sufficient documentation of bilateral synchronous orbicularis oculi spasms inducing eyelid narrowing or closure together with increased blinking rate and/or recognition of a sensory trick, according to the clinical guidelines [2]. Patients with insufficient clinical information to evaluate the diagnosis and patients with no evidence of blepharospasm were excluded (Figure 1). Patients with blepharospasm combined with other movement disorders, patients with other acquired etiology than lesions (e.g. drug-induced), and patients with onset of dystonia elsewhere in the body were also excluded to limit the analyses to blepharospasm with uncertain etiology prior to brain imaging only (Figure 1). However, patients with blepharospasm and spread of dystonia to other body regions later in the disease course or patients with blepharospasm combined with other neurological features than movement disorders were not excluded [1,16].

The study protocol was approved by the Hospital District of Southwest Finland and the need for separate ethical committee evaluation or patient consent were waived because of the retrospective nature of the study, according to the institutional and national rules and regulations. The study was conducted according to the principles of the Declaration of Helsinki.

### Clinical data

Clinical features were documented following previously published work [4,16], including orbicularis oculi spasms, increased blinking rate, sensory trick (geste antagoniste), pretarsal blepharospasm, spread of dystonia to other body regions, temporal evolution and unilateral/asymmetric onset of blepharospasm. When assessing the spread of dystonia, other body regions affected were categorized to the lower cranial region, cervical region, larynx, trunk, upper limbs, and lower limbs, and distribution of dystonia was classified to focal, segmental, multifocal and generalized, according to the classification of dystonia [1]. In addition, other neurological features at the onset of blepharospasm were documented [11,16].

Remission of blepharospasm, considered as an uncommon feature in the earlier literature [4] as well as acute/subacute onset (blepharospasm reaching peak severity within 3 months from the symptoms onset), and other neurological features at the onset of blepharospasm [11,16], were considered as atypical features for idiopathic blepharospasm in this study.

The date of blepharospasm diagnosis and date of last clinical follow-up or death were documented for each patient to calculate the prevalence and annual incidence of blepharospasm, and the duration of the retrospective clinical follow-up in this study. The prevalence and annual incidence of blepharospasm were then defined by calculating the mean annual incidence and prevalence over the 26-year time (years 1996–2021) using the annual population count in the area for each year from Statistics Finland (https://stat.fi/index_en.html).

### Neuroimaging data

From patients with blepharospasm with uncertain etiology prior to brain imaging, all clinical head CT and MRI scans were re-evaluated by the investigators to detect focal brain lesions, including all intracranial intraparenchymal or compressive lesions, such as infarctions, intracerebral hemorrhages, cysts or tumors, but not atrophy or vascular degeneration. Only lesions preceding the onset of blepharospasm were assessed as potentially causal for blepharospasm.

### Lesion-induced blepharospasm

In patients with focal brain lesions preceding the onset of blepharospasm, causality was evaluated by an in-depth review of all the available clinical and imaging data by E.M. and J.J. As there are currently no established guidelines for the diagnosis of any lesion-induced movement disorders [17], the evaluation was performed case-by-case by the investigators, leveraging information from the recently published systematic review of all published cases of lesion-induced dystonia [16]. First, lesions interpreted to be causal for blepharospasm were required to have a logical temporal association with the onset of symptoms, i.e. lesion preceding the symptom onset with no more than 5.5 years delay (which is the maximum reported delay in lesion-induced dystonia) [16]. Second, the lesion laterality was required to be neuroanatomically logical with lateralizing symptoms. However, we did not make restrictions based on brain regions affected by the lesions, because lesions causing dystonia have been shown to occur in heterogeneous locations [16]. Third, as incidental lesions are not rare [18], at least some additional supportive information linking the lesion to the symptoms was required to diagnose lesion-induced dystonia, i.e. patients with typical, symmetric, slowly developing blepharospasm without a close temporal association to a lesion discovered on brain imaging were not considered as lesion-induced cases. Finally, the identified lesion was considered more likely etiology for blepharospasm than other possible etiologies, including idiopathic condition.

### Blepharospasm with and without atypical features

After testing group differences between lesion-induced and idiopathic blepharospasm, imaging findings between cases with (at least one atypical feature) and without atypical features were tested post-hoc to investigate whether neuroimaging is needed in patients without atypical features to capture lesion-induced cases from this patient population.

### Data analysis

All statistical analyses were performed using IBM SPSS Statistics (version 29, IBM Corp., New York, USA). The assumption of normality was evaluated visually from histograms, together with Shapiro-Wilk tests. Clinical characteristics between lesion-induced and idiopathic blepharospasm, and imaging findings between cases with and without atypical features, were tested using Mann-Whitney U test or Fisher Exact test, as appropriate. *P*-values < 0.05 were considered significant.

## Results

Our search identified 79 patients with blepharospasm that were confirmed based on the review of the clinical records (Figure 1). Of these patients, 17 (21.5%) had blepharospasm combined with other movement disorders (Parkinson’s disease n = 9, progressive supranuclear palsy n = 5, multiple system atrophy n = 1, dementia of Lewy bodies n = 1, spinocerebellar ataxia n = 1), three (3.8%) had drug-induced blepharospasm, and two (2.5%) had cervical dystonia with later spread of dystonia to the upper cranial region, and were therefore excluded, leaving 57 patients (72.2%) with blepharospasm with uncertain etiology prior to brain imaging (Figure 1). Demographic and clinical features of these 57 patients are shown in Table 1.

**Table 1:** Demographic and clinical characteristics of patients with blepharospasm with uncertain etiology.


DEMOGRAPHIC FEATURES	

Number of patients	57

Age at blepharospasm diagnosis, mean (SD)	63.0 (10.8)

Sex (F), *n*(%)	41 (71.9%)

Duration of follow-up in years, median (range)	9.0 (0.17–25.9)

**CLINICAL FEATURES**	

Orbicularis oculi spasms, *n*(%)	57 (100%)

Increased blinking rate, *n*(%)	55 (96.5%)

Sensory trick (geste antagoniste), *n*(%)	7 (12.3%)

Pretarsal blepharospasm, *n*(%)	4 (7.0%)

Spread of dystonia to other body regions, *n*(%)	

No spread (focal dystonia), *n*(%)	31 (54.4%)

Spread, *n*(%)	26 (45.6%)

Segmental, *n*(%)	18/26 (69.2%)

Multifocal, *n*(%)	7/26 (26.9%)

Generalized, *n*(%)	1/26 (3.8%)

Unilateral/asymmetric onset of blepharospasm,*n*(%)	8 (14.0%)

Acute/subacute onset of blepharospasm, *n* (%)	2 (3.5%)

Other neurological features at the onset of blepharospasm, *n* (%)	8 (14.0%)

Remission of blepharospasm, *n*(%)	4 (7.0%)

**BRAIN IMAGING**	

Brain imaging with CT/MRI, *n*(%)	39 (68.4%)

Focal brain lesions^1^, *n* (%)	7/39 (17.9%)

Causal lesions, *n* (%)	4/39 (10.3%)


^1^Any focal brain lesions before the onset of blepharospasm.

The population-based prevalence and annual incidence of blepharospasm (all patients with blepharospasm) were 60.0 per million and 6.6 per million, and of blepharospasm with uncertain etiology prior to brain imaging were 50.4 per million and 4.7 per million, respectively.

### Lesion-induced blepharospasm

Of the 57 patients with blepharospasm with uncertain etiology prior to brain imaging, 39 (68.4%) underwent brain imaging using head MRI or CT. Seven (17.9%) of the scanned patients had focal brain lesions preceding the onset of blepharospasm, and four (10.3%) of them were considered to have lesion-induced blepharospasm (Table 1, Supplementary Table 1). These four cases included three patients with ischemic strokes in heterogeneous locations and one with meningioma compressing the cerebellum (Supplementary Figure 1). The three remaining patients with focal brain lesions in imaging and the 50 patients without lesions were considered idiopathic.

The percentage of patients with lesion-induced blepharospasm was 7.0% (4/57) of patients with blepharospasm with uncertain etiology prior to brain imaging and 5.0% (4/79) of all patients with blepharospasm. The population-based prevalence of lesion-induced blepharospasm was 2.5 per million and the annual incidence was 0.3 per million. Patients with lesion-induced blepharospasm had significantly more often atypical features, including acute/subacute onset, other neurological features or remission of blepharospasm, compared to patients with idiopathic blepharospasm (Fisher Exact test, *P* < 0.05, Table 2).

**Table 2:** Clinical characteristics of patients with lesion-induced and idiopathic blepharospasm.


	LESION-INDUCED	IDIOPATHIC	*P*-VALUE

	*N* = 4	*N* = 53	

Age at blepharospasm diagnosis, median (range)	66.5 (65–71)	64.0 (29–87)	0.444

Sex (F), *n* (%)	3 (75%)	38 (71.7%)	1.000

Brain imaging, *n* (%)	4 (100%)	35 (66%)	–

Orbicularis oculi spasms, *n* (%)	4 (100%)	53 (100%)	1.000

Increased blinking rate, n (%)	4 (100%)	51 (96.2%)	1.000

Sensory trick (geste antagoniste), n (%)	0 (0%)	7 (13.2%)	1.000

Pretarsal blepharospasm, n (%)	0 (0%)	4 (7.5%)	1.000

Spread of dystonia to other body regions, *n* (%)	1 (25%)	25 (47.2%)	0.617

Unilateral/asymmetric onset of blepharospasm, *n* (%)	4 (100%)	4 (7.5%)	**<0.001**

Acute/subacute onset of blepharospasm, *n* (%)	2 (50%)	0	**0.004**

Other neurological features at the onset of blepharospasm, *n* (%)	4 (100%)	4 (7.5%)	**<0.001**

Remission of blepharospasm, *n* (%)	2 (50%)	2 (3.8%)	**0.021**


### Blepharospasm with vs. without atypical features

Ten patients (17.5%) had at least one atypical clinical feature and were all scanned with head MRI or CT. Of the 47 patients without atypical features, 29 (61.7%) were scanned. Lesion-induced blepharospasm was significantly more common in patients with atypical features compared to patients without, as lesion-induced etiology was identified in 40.0% of patients with and none of patients without atypical features (Fisher Exact test, *P* = 0.003, Table 3).

**Table 3:** Imaging findings in patients with atypical and typical blepharospasm.


	ATYPICAL BLEPHAROSPASM^1^	TYPICAL BLEPHAROSPASM^1^	*P*-VALUE

	*N* = 10	*N* = 47	

Brain imaging, *n* (%)	10 (100%)	29 (61.7%)	**0.022**

Focal brain lesions^2^, *n* (%)	4 (40.0%)	3 (10.3%)	0.057

Causal lesions, *n* (%)	4 (40.0%)	0	**0.003**


^1^Acute/subacute onset of blepharospasm and/or other neurological features at the onset of blepharospasm and/or complete remission of blepharospasm. ^2^Any focal brain lesions before the onset of blepharospasm.

## Discussion

There are several important findings in this study. First, our results show that lesion-induced blepharospasm is likely more common than thought previously, affecting 7.0% of patients with blepharospasm with uncertain etiology prior to brain imaging, corresponding to a population-based prevalence of 2.5 per million and annual incidence of 0.3 per million. Second, causal lesion locations were heterogeneous, aligning with observations in other lesion-induced dystonias [16]. Third, all lesion-induced cases showed atypical features, suggesting that routine structural brain imaging in blepharospasm is needed only in patients with atypical features to identify lesion-induced cases.

The percentage of lesion-induced cases was markedly higher in our sample compared to earlier studies (0.4–1.6%) (12–14). The substantially higher percentage may be related to more frequent use of neuroimaging in our cohort (68.4% of the patients were scanned compared to 15.6% in an earlier study [13]) and our focus on patients with blepharospasm with presumed idiopathic or uncertain etiology prior to brain imaging only. The other two studies investigating this topic did not report the proportion of the scanned patients [12,14], aligning with the clinical guidelines that generally do not recommend routine neuroimaging in patients with isolated blepharospasm without atypical features [15]. Accordingly, the percentage of lesion-induced cases from patients with blepharospasm with uncertain etiology who had undergone brain imaging in the earlier study by Khooshnoodi et al. (11.5%) was similar compared to that in our study (10.3%) [13]. Our study also included a thorough review of the medical records specifically to identify lesion-induced cases instead of relying on medical records or diagnosis codes alone, which may also explain the higher number of identified cases in the present study. In addition, there may be differences in the studied populations. For example, one of the earlier studies included a cohort of blepharospasm patients from the department of ophthalmology only [12], which is likely to favor including patients without atypical features that do not require neurological differential work up, whereas our study included all patients from our hospital from both neurology and ophthalmology.

In our re-review of the medical records of the patients identified through the systematic search of the hospital database, we identified a substantial number of incorrect/inaccurate diagnoses (most of which were corrected during follow-up by their treating physician), indicating that studying epidemiology of blepharospasm based on diagnosis code registries alone may not be reliable. Moreover, only one of the four lesion-induced cases in our study were recognized as such by their treating physicians. However, it should be noted that despite the careful review of the medical records, our study was still retrospective and would miss cases who are not recognized in the primary care or do not seek treatment, leading to underestimation, rather than overestimation, of the prevalence and incidence of lesion-induced blepharospasm. All patients with lesion-induced blepharospasm showed atypical features, supporting the view that neuroimaging is not needed in patients with isolated blepharospasm without atypical features [15]. Our findings are supported by cases from a recent systematic review of lesion-induced dystonias [16], which included 44 cases with lesion-induced blepharospasm. Of these 44 cases, only two cases with a sufficient description of the neurological exam findings had possible lesion-induced blepharospasm without any atypical features. Thus, lesion-induced isolated blepharospasm without atypical features may not be impossible but is, per minimum, exceedingly rare based on the results of the current study and available literature. It should be noted that three of our patients showed brain lesions without any apparent link to patient’s blepharospasm and were thus considered as incidental lesions, which are also common in healthy populations [18], but their role in the pathogenesis of blepharospasm cannot be definitely excluded [11].

As in dystonia in general, the pathophysiology of secondary blepharospasm is not yet fully understood [11,15]. The causal lesions in our cases were in heterogeneous locations with little overlap, aligning with the results of a recent systematic review of all published cases of lesion-induced dystonia (n = 359) [16]. The basal ganglia, the most common anatomical location in lesion-induced dystonia (46.2%) [16], was involved in two of the four cases. The heterogeneity in lesion locations is likely explained by recent findings in several different movement disorders applying lesion network mapping, showing that causal lesions localize to brain networks rather than individual anatomical structures [19,20,21,22,23]. In cervical dystonia, the causal lesions localized to a brain network including the cerebellum, thalamus, basal ganglia and sensorimotor cortices, aligning with the network model of dystonia, which may also apply to blepharospasm [19]. However, the brain network underlying blepharospasm is yet to be identified, but this may become feasible along with increasing number of published cases of lesion-induced blepharospasm, such as described here. Characterizing the network underlying blepharospasm could be a valuable resource for evaluating the causality of lesions in patients with blepharospasm. For example, involvement of the brain network identified based on lesions causing limb ataxia was shown to predict development of limb ataxia in an independent sample of stroke patients [23].

One of the four patients with lesion-induced blepharospasm showed delayed-onset of symptoms (2.5 years), which again aligns with the phenomenology of lesion-induced dystonia described in the literature [16]. Similarly, an older well-documented case series of lesion-induced blepharospasm by Jankovic & Patel reported latencies ranging from two days up to 3 years [24]. Interestingly, the prognosis of lesion-induced blepharospasm seems relatively favorable as two of the four patients remitted, which was significantly more common than in patients with idiopathic blepharospasm (3.8%, 2/53). It should be noted that remission of dystonia has previously been considered to be a sign of functional dystonia, but this view has more recently been revoked as there are well-documented cases of dystonia who have eventually remitted [25]. Our findings also align with one of the earlier cohort studies reporting remission of the symptoms in two of six patients with lesion-induced blepharospasm [12]. Based on previous research, response to botulinum toxin injections seems comparable between idiopathic and acquired blepharospasm [26].

This study has several limitations that should be considered when interpreting the results. First, due to the retrospective study design, the diagnoses couldn’t be confirmed by a clinical examination by the investigators, and we lacked systematically recorded data from non-motor features, such as photophobia [27]. Second, despite the extended time window (26 years) for the data search, the study sample was still relatively small. However, as the Finnish healthcare system is regionally organized, the prevalence and incidence can be considered as population-based estimates. Third, although use of brain imaging was much more common in our sample than in the previous studies (most likely reflecting the good availability and frequent use of imaging in Finnish healthcare system), still more than a third of the patients without atypical features were not scanned. Thus, missing lesion-induced cases because of lack of imaging data, which could lead to overestimation of the group difference between patients with and without atypical features, cannot be excluded. However, we feel this is unlikely as none of the patients with typical blepharospasm, who were scanned, were shown to have causal lesions. Finally, as established guidelines for the diagnosis of lesion-induced blepharospasm are lacking [17], the diagnoses of lesion-induced blepharospasm in this study were based on case-by-case evaluation using a criteria crafted based on the available literature for the purposes of this study.

To conclude, this study shows that lesion-induced etiology of blepharospasm is not as rare as previously thought, affecting 7.0% of patients with blepharospasm with uncertain etiology.

## Additional Files

The additional files for this article can be found as follows:

10.5334/tohm.1025.s1 Supplementary Figure 1. Lesions of patients with lesion-induced blepharospasm (panel A, patients 1–4) and patients with blepharospasm and incidental lesions (panel B, patients 5–7). Lesions are demonstrated by white arrows.

10.5334/tohm.1025.s2 Supplementary Table 1. Cases of blepharospasm with brain lesions preceding the onset of blepharospasm.

## Financial disclosures

E.M. was supported by personal grants from the Finnish Parkinson Foundation, Maire Taponen Foundation and Turku University Foundation.

R.H.: No personal grants.

D.C. was supported by the Dystonia Medical Research Foundation under award number DMRF-BCAD-2023-1.

J.J. reports research grants from the Finnish Research Council, Finnish Medical Foundation, Sigrid Juselius Foundation, Finnish Foundation for Alcohol Studies, University of Turku and Turku University Hospital; conference travel support from Insightec, Abbvie and Abbott; consulting for Teva Finland, Summaryx and Adamant Health; lecturer honoraria from Nordic Infucare, Lundbeck and Novartis; acting as an advisory board member for Teva Finland.
