# Supplementary figures and images for: Lesion-Induced Blepharospasm: Epidemiology and Clinical Characteristics
Source: Tremor Other Hyperkinet Mov (N Y). 2025 Jun 9;15:25. doi: 10.5334/tohm.1025 (PMC12164745; doi:10.5334/tohm.1025)

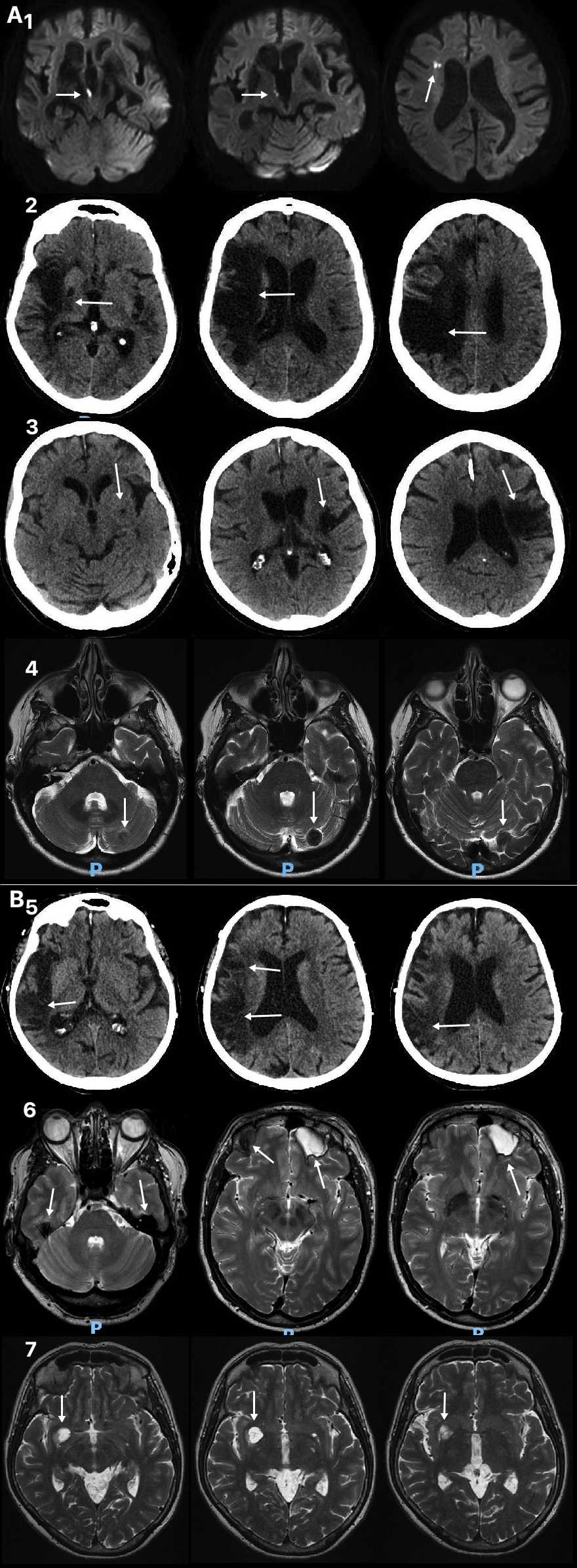

Supplement: Supplementary Figure 1. — Lesions of patients with lesion-induced blepharospasm (panel A, patients 1–4) and patients with blepharospasm and incidental lesions (panel B, patients 5–7). Lesions are demonstrated by white arrows. [file tohm-15-1-1025-s1.tiff]
