# Supplementary material for: Lesion-Induced Blepharospasm: Epidemiology and Clinical Characteristics
Source: Tremor Other Hyperkinet Mov (N Y). 2025 Jun 9;15:25. doi: 10.5334/tohm.1025 (PMC12164745; doi:10.5334/tohm.1025)
Supplement: Supplementary Table 1. — Cases of blepharospasm with brain lesions preceding the onset of blepharospasm. [file tohm-15-1-1025-s2.pdf]

Supplementary Table 1. Cases of blepharospasm with brain lesions preceding the onset of blepharospasm. Patients 1-4 were considered as cases of lesion-induced blepharospasm and patients 5-7 to have incidental brain lesions. The mean (SD, range) age of the patients was 64.0 (8.1, 48-73) years with 7.7 (3.2, 3-11) years follow up, including two male and five female patients. The corresponding lesions are presented in Supplementary Figure 1.

**Causal lesions**	**Lesion type**	**Lesion locations**	**Latency**	**Blepharospasm onset**	**Other symptoms at blepharospasm onset**	**Evolution after onset**	**Treatments (response)**	**Outcome**	**Lesion-induced**
Patient 1	Ischemic stroke	Mesencephalon, thalamus, centrum semiovale R	< 2mo	Left-predominant	Partial oculomotor paresis R, hemiextinction L	Progressive	Botulinum toxin (+)	Continued until the death of the patient	Yes
Patient 2	Ischemic stroke	MCA and ACA territories R	Acute	Left (unilateral)	Hemiparesis L	Progressive to bilateral	Botulinum toxin (+)	Continued until the death of the patient	Yes
Patient 3	Ischemic stroke	Frontal cortex and frontal white matter, putamen L	2.5y	Right-predominant	Hemiparesis R	Improved	No treatments	Remitted	Yes
Patient 4	Meningioma	Cerebellum L	N.a.	Left-predominant, worsened when laying down	Lower limb paresthesia L, daily headaches	Stable	Clonazepam (+)	Remitted	Yes
**Incidental lesions**	**Lesion type**	**Lesion locations**	**Latency**	**Blepharospasm onset**	**Other symptoms at blepharospasm onset**	**Evolution**	**Treatments (response)**	**Outcome**	**Lesion-induced**
Patient 5	Ischemic stroke	Temporal cortex, parietal cortex R	6y	Symmetric	None	Progressive	Botulinum toxin (+)	Continued until death of the patient	No
Patient 6	Traumatic brain injury	Frontal cortex, temporal cortex R+L	7y	Symmetric	None	Progressive	Botulinum toxin (+)	Continued until the end of the follow-up	No
Patient 7	Cyst	Putamen R	N.a.	Symmetric	None	Progressive	Botulinum toxin (+)	Continued until the end of the follow-up	No

F = female. M = male. R = right. L = left. MCA = middle cerebral artery. ACA = anterior cerebral artery. N.a. = not applicable. + = good response.
